# Inositol-Requiring Enzyme 1 Alpha Endoribonuclease Specific Inhibitor STF-083010 Alleviates Carbon Tetrachloride Induced Liver Injury and Liver Fibrosis in Mice

**DOI:** 10.3389/fphar.2018.01344

**Published:** 2018-11-27

**Authors:** Qian-Qian Chen, Cheng Zhang, Ming-Qiang Qin, Jian Li, Hua Wang, De-Xiang Xu, Jian-Qing Wang

**Affiliations:** ^1^The Fourth Affiliated Hospital, Anhui Medical University, Hefei, China; ^2^The Second Affiliated Hospital, Anhui Medical University, Hefei, China; ^3^Department of Toxicology, Anhui Medical University, Hefei, China

**Keywords:** inositol-requiring enzyme 1 alpha, STF-083010, miR-122, hepatocyte death, liver fibrosis

## Abstract

Accumulating data demonstrated that hepatic endoplasmic reticulum (ER) stress was involved in the pathogenesis of liver fibrosis. Long-term chronic hepatocyte death contributed to liver fibrosis initiation and progression. Previous researches reported that ER stress sensor inositol-requiring enzyme 1 alpha (IRE1α) was first activated in the process of liver fibrosis. STF-083010 was an IRE1α RNase specific inhibitor. This study aimed to explore the effects of STF-083010 on carbon tetrachloride (CCl_4_)-induced liver injury and subsequent liver fibrosis. Mice were intraperitoneally (i.p.) injected with CCl_4_ (0.15 ml/kg) for 8 weeks. In STF-083010+CCl_4_ group, mice were injected with STF-083010 (30 mg/kg, i.p.), twice a week, beginning from the 6th week after CCl_4_ injection. CCl_4_ treatment markedly enhanced the levels of serum ALT, TBIL, DBIL and TBA, and STF-083010 had obviously extenuated CCl_4_-induced exaltation of ALT, DBIL, and TBA levels. CCl_4_-induced hepatic hydroxyproline and collagen I, major indicators of liver fibrosis, were alleviated by STF-083010. Additionally, CCl_4_-induced α-smooth muscle actin, a marker for hepatic stellate cells activation, was obviously attenuated in STF-083010-treated mice. Moreover, CCl_4_-induced upregulation of inflammatory cytokines was suppressed by STF-083010. Mechanistic exploration found that hepatic miR-122 was downregulated in CCl_4_-treated mice. Hepatic MCP1, CTGF, P4HA1, Col1α1, and Mmp9, target genes of miR-122, were upregulated in CCl_4_-treated mice. Interestingly, STF-083010 reversed CCl_4_-induced hepatic miR-122 downregulation. Correspondingly, STF-083010 inhibited CCl_4_-induced upregulation of miR-122 target genes. This study provides partial evidence that STF-083010 alleviated CCl_4_-induced liver injury and thus protected against liver fibrosis associated with hepatic miR-122.

## Introduction

Liver fibrosis is a reversible wound-healing response in the liver to cellular injury, reflecting the balance between liver repair and scar tissue formation. Liver fibrogenesis is driven by transdifferentation of hepatic stellate cells (HSCs) to α-smooth muscle actin (SMA)-positive myofibroblasts, which represents excessive accumulation of extracellular matrix (ECM) (Kendall and Feghali-Bostwick, 2014; Zhao et al., 2017). It is generally believed that HSCs activation is the main cell event during the progression of liver fibrosis (Chen et al., 2015). Liver fibrosis usually occurs in response to chronic inflammation caused by viral infection, toxic substances, alcohol abuse, cholestasis, and fatty deposition of the liver (Kramann et al., 2013). So far, there is no specific drug to reverse the process of liver fibrosis. Therefore, it is important to elucidate the pathogenesis of hepatic fibrosis for the prevention and treatment.

It has been found that hepatic endoplasmic reticulum (ER) stress plays an important role in many liver diseases and becomes a new target for drug discovery (Rivas et al., 2015). The ER is responsible for such tasks as protein synthesis, folding, assembly and shipping in eukaryotic cells (Kim et al., 2016). Homeostatic regulation of the ER is under the control of three evolutionary conserved pathways: inositol-requiring enzyme 1 alpha (IRE1α), double-stranded RNA-activated kinase (PKR)-like ER kinase (PERK) and activating transcription factor 6 (ATF6) (Malhi and Kaufman, 2011; Kropski and Blackwell, 2018). IRE1α as an executor of cell fate determination under ER stress, it also affects the fate of ATF6 and PERK signaling pathways (Lin et al., 2007; Chen and Brandizzi, 2013). The study found that IRE1α signaling pathway was first activated after giving the mice a single carbon tetrachloride (CCl_4_) stimulus, (Lin et al., 2007; Hetz et al., 2011) and one of our previous study found that IRE1α signaling was first activated in CCl_4_-induced liver fibrosis (Wang et al., 2013). The activated IRE1α has dual activities of protein kinase and RNase, which its oligomerization-mediated trans-autophosphorylation in turn leads to IRE1α RNase activation (Han et al., 2009). In recent years, some studies have shown that IRE1α RNase activation governs cell fate through selectively cleaving mRNA and microRNAs (miRNAs) (Hassler et al., 2012; Upton et al., 2012; Moore and Hollien, 2015). STF-083010 is identified as a new chemical molecule that selectively inhibits IRE1α RNase activity and degradation of specific mRNA without affecting its kinase activity. Moreover, it has shown a potential to effectively control ER stress-induced disorders (Papandreou et al., 2011; Liu et al., 2018). Thus, it is interesting to explore the effect of IRE1α RNase specific inhibitor STF-083010 on hepatic fibrosis.

This study aimed to explore the effect of STF-083010 on CCl_4_-induced liver injury and subsequent liver fibrosis in mice. Our results found that IRE1α RNase specific inhibitor STF-083010 protected against CCl_4_-induced hepatocyte death. The present study provided partial evidence that hepatic IRE1α RNase was involved in CCl_4_-induced liver fibrosis through regulating hepatic miR-122. The research results will provide a theoretical basis for discovering new effective therapeutic targets and reversing hepatic fibrosis.

## Materials and Methods

### Chemicals and Reagents

CCl_4_, fast green FCF, direct red 80 (sirius red) and anti-α-SMA monoclonal antibody were purchased from Sigma Chemical Co. (St. Louis, MO, United States). STF-083010 was from MedChemexpress (New Jersey, United States). Anti-p-IRE1α/IRE1α antibodies, anti-(glucose-regulated protein, GRP78) antibody, TRIzol^®^reagent, Chemiluminescence (ECL) were from Thermo Fisher Scientific Inc (Rockford, IL, United States), anti-α-tubulin antibody and anti-3-nitrotyrosine (3-NT) antibody were from Santa Cruz Biotechnologies (Santa Cruz, CA, United States). RNase-free DNase and AMV were purchased from Promega Corporation (Madison, WI, United States). LightCycler480^®^SYBR Green I Master was from Roche Diagnostics GmbH (Mannheim, Germany). All primers were synthesized by Invitrogen Trading (Shanghai) Co., Ltd. All other reagents were purchased from Sigma Chemical Co. (St. Louis, MO, United States) if not otherwise stated.

### Animals and Treatments

Male ICR mice (6–8w, 24–26 g) were from Beijing Vital River (China). The animal experimental procedures were reviewed and approved by the Animal Ethical Committee of Anhui Medical University (Permit Number: LLSC20140047). The mice were allowed free access to water and food and were maintained on a suitable environment [temperature (20–25°C) and humidity (50 ± 5%)] for a period of 1 week before use. Mice were randomly divided into four groups. In CCl_4_ alone, mice were intraperitoneally (i.p.) injected with CCl_4_ (0.15 ml/kg, 10% soluble in corn oil, twice a week) for 8 weeks. In control group, mice were normal feeding with the dissolvent injection. In STF-083010+CCl_4_ group, mice were injected with STF-083010 (30 mg/kg, i.p.), twice a week, beginning from the 6th week after CCl_4_ injection. In STF-083010 alone group, mice were only i.p. injected with STF-083010. All mice were sacrificed after 8 weeks with CCl_4_ treatment, blood and livers were collected for the experiment. Serum was isolated for measurement of biochemical parameters. Some liver tissues were fixed in 4% paraformaldehyde solution for histological examination and immunohistochemistry, and others were frozen immediately in lipid nitrogen for real-time RT-PCR and Western blot.

### Biochemical Assays

The serum levels of alanine aminotransferase (ALT), direct bilirubin (DBIL), and total bilirubin (TBIL) were detected by automatic biochemical analyzer. Total bile acid (TBA) in serum and malondialdehyde (MDA) in tissue (refer to Supplementary Figure S1) were measured by commercially available assay kits under manufacturer’s instructions.

### Histology

The isolated liver tissue was fixed in freshly prepared paraformaldehyde solution (4%) for 24 h and then embedded in paraffin under standard procedures. The tissue stored in the slide was cut into 5 μm thick, and then stained with hematoxylin and eosin (H&E) for further analysis. The inflammatory cells were counted in 12 randomly selected fields from each slide (magnification × 400). The areas of hepatic necrosis were measured as the average of all necrotic fields within each slide.

### Determination of Liver Fibrosis

Hepatic fibrosis was determined with masson’s trichrome staining and sirius red staining. Briefly, sections were deparaffinized with xylene and hydrated with gradient ethanol. In masson’s trichrome staining, hepatic tissue sections were stained with Mayer hematoxylin for 3 min, magenta stained for 10 min and aniline blue stained for 5 min, and then processed to neutral gum seal. In sirius red staining, hepatic tissue sections were immersed in saturated picric acid solution (containing 0.1% Fast Green FCF and 0.1% Direct Red 80) for 2 h in the dark. A light microscope equipped with CCD digital camera (DP-80, Olympus) captures the morphology of collagen fibers. Morphometric analysis for liver fibrosis quantification was measured using whole tissue low-power images every mouse (magnification ×100). Percentages of collagen accumulation areas were measured by using NIH Image J software^1^.

### Hepatic Hydroxyproline Examination

Hepatic hydroxyproline (Hyp) content was measured using commercially assay kits under manufacturer’s instructions (Reddy and Enwemeka, 1996).

### Isolation of Total RNA and Real-Time RT-PCR

Hepatic total RNA was extracted using TRIzol. The purity of total RNA was evaluated based on the absorbance ratio at 260 and 280 nm. RNase-free DNase-treated total RNA was reverse-transcribed with AMV in Biometra T Gradient Thermocycler. Real-time RT-PCR was carried out with a LightCycler^®^480 SYBR Green I kit by using gene-specific primers as listed in Table 1. The amplification reactions were performed on a LightCycler^®^480 Instrument (Roche Diagnostics GmbH, Mannheim, Germany) with a pre-incubation step (95°C for 10 min) and 45 cycles of a three-step PCR (95°C for 15 s, 60°C for 15 s, 72°C for 20 s). The comparative C_T_-method was used to determine the amount of target genes (Hoebeeck et al., 2007; Ferlini and Rimessi, 2012), normalized to an endogenous reference and relative to a calibrator (2^-ΔΔCt^) using the LightCycler 480 software (Roche, version 1.5.0). All RT-PCR experiments were carried out in triplicate.

**Table 1:** Oligonucleotide sequence of primers for real-time RT-PCR.

Genes	Forward primers (5′–3′)	Reverse primers (5′–3′)
18S	GTA ACC CGT TGA ACC CCA TT	CCA TCC AAT CGG TAG TAG CG
TGF-β1	CGG GAA GCA GTG CCC GAA CC	GGG GGT CAG CAG CCG GTT AC
IL-1β	GCC TCG TGC TGT CGG ACC CAT AT	TCC TTT GAG GCC CAA GGC CAC A
MCP1	GGC TGG AGA GCT ACA AGA GG	GGT CAG CAC AGA CCT CTC TC
IL-6	AGACAAAGCCAGAGTCCTTCAGAGA	GCC ACT CCT TCT GTG ACT CCA GC
α-SMA	GAG ACT CTC TTC CAG CCA TCT T	TGA TCT CCT TCT GCA TCC TGT C
XBP1s	CTG AGT CCG AAT CAG GTG CAG	GTC CAT GGG AAG ATG TTC TGG
XBP1t	TGG CCG GGT CTG CTG AGT CCG	GTC CAT GGG AAG ATG TTC TGG
Col1α1	CAA TGG CAC GGC TGT GTG CG	AGC ACT CGC CCT CCC GTC TT
Col1α2	CTC ATA CAG CCG CGC CCA GG	AGC AGG CGC ATG AAG GCG AG
Mmp9	CGG CAC GCC TTG GTG TAG CA	AGG CAG AGT AGG AGC GGC CC
P4HA1	CCA CAG CAG AGG AAT TAC AG	ACA CTA GCT CCA ACT TCA GG
CTGF	ACC CAA CTA TGA TTA GAG CC	TTG CCC TTC TTA ATG TTC TC

### Stem-Loop RT-PCR for MiR-122

For detection of miR-122, total RNA was reverse-transcribed using stem-loop primer (RT miR-122: 5′- GTC GTA TCC AGT GCA GGG TCC GAG GTA TTC GCA CTG GAT ACG ACC AAA C-3′; RT U6: 5′-AAC GCT TCA CGA ATT TGC GT-3′). Specific oligonucleotide primers were used for miR-122 and U6 genes: miR-122 F: 5′-TGG AGT GTG ACA ATG GTG TT-3′ and R: 5′-CCA GTG CAG GGT CCG AGG T-3′; U6: F: 5′-CTC GCT TCG GCA GCA CA-3′, and R: 5′-AAC GCT TCA CGA ATT TGC GT-3′ (Kazantseva et al., 2015). RT reaction was carried out in Biometra T Gradient Thermocycler (16°C for 30 min, 42°C for 30 min, 85°C for 5 min and taken out the sample at 4°C). The amplification reactions were carried out and the expression levels of miR-122 were quantified as described above. MiR-122 was normalized with U6 snRNA.

### Immunoblots

Hepatic lysate was prepared using homogenizing 50-80 mg liver tissue in lysis buffer supplemented with a cocktail of protease inhibitors. The concentration of protein was determined by the bicinchoninic acid (BCA) protein assay (Pierce, Rockford, IL, United States). For immunoblot, same amount of protein boiled with 2× Laemmli denaturing and loading buffer was separated electrophoretically by SDS-PAGE and transferred to a polyvinylidene fluoride membrane. The membranes were incubated for 2 h with following antibodies: α-SMA (1:5000), p-IRE1α (1:2000) or IRE1α (1:2000) and GRP78 (1:2000). Alpha-tubulin was used as a loading control. The membranes were incubated with either goat anti-mouse or goat anti-rabbit IgG antibodies for 2 h. The membranes were imaged by signal development using an ECL detection kit.

### Immunohistochemistry

Liver tissue sections were deparaffinized and subsequently hydrated in gradient ethanol. After quenching endogenous peroxidase, tissue sections were incubated with anti-α-SMA monoclonal antibody (1:1000) or anti-3-nitrotyrosine (3-NT) antibody (1:200) at 4°C overnight. The color reaction was developed with Horseradish Peroxidase (HRP)-linked polymer detection system and counterstaining with hematoxylin. Alpha-SMA positive area was analyzed in each slide (magnification ×100). The percentages of α-SMA positive area were quantified using NIH Image J software in six mice from each group.

### Statistical Analysis

All data were expressed as means ± SEM. ANOVA and the Student-Newmann-Keuls *post hoc* test were used to determine differences among different groups. A *P*-value of 0.05 was set as the threshold for statistical significance.

## Results

### STF-083010 Attenuates CCl_4_-Induced Hepatic IRE1α RNase Activation in Mice

**FIGURE 1:**
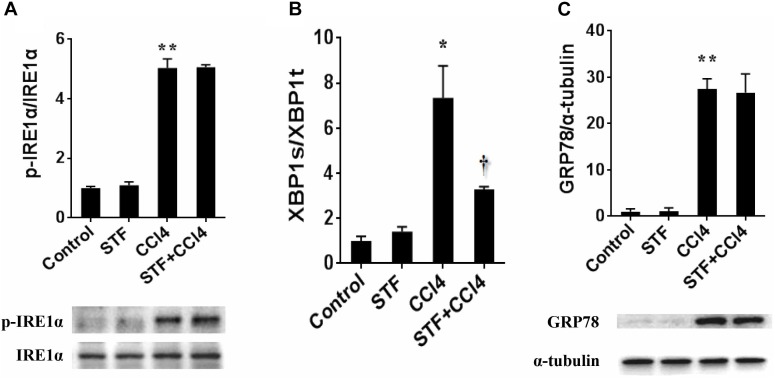
The effect of STF-083010 (STF) on IRE1α RNase activation in CCl_4_-induced liver fibrosis in mice. Mice were i.p. injected with CCl_4_ (0.15 ml/kg, twice per week). In STF-083010+CCl_4_ group, mice were injected with STF-083010 (30 mg/kg, i.p.), twice per week, beginning from the 6th week after CCl_4_ injection. All mice were sacrificed after 8 weeks with CCl_4_ treatment. **(A,C)** Hepatic p-IRE1α, IRE1α and GRP78 were measured using immunoblot. A representative protein band for p-IRE1α or GRP78 (upper panel) and IRE1α or α-tubulin (lower panel) was shown. All experiments were repeated for four times. Quantitative analyses of scanning densitometry for p-IRE1α/IRE1α and GRP78/α-tubulin on six samples from six different mice were carried out. **(B)** Hepatic XBP1s and XBP1t mRNAs were detected using real-time RT-PCR, XBP1s/XBP1t were analyzed. All data were expressed as means ± SEM (*n* = 6). ^∗^*P* < 0.05, ^∗∗^*P* < 0.01 versus control group. ^†^*P* < 0.05 versus CCl_4_ group.

The roles of IRE1α RNase activation in CCl_4_-induced liver fibrosis in mice were shown in Figure 1. As expected, the phosphorylation level of hepatic IRE1α was apparently activated in CCl_4_-treated mice, but STF-083010 unaffected its level in CCl_4_-treated fibrotic mice (Figure 1A). Further study indicated that ratios of spliced XBP1 (XBP1s) to total XBP1 (XBP1t) transcripts, an indicator of IRE1α RNase activity, were obviously elevated in CCl_4_-induced liver fibrosis in mice. Interestingly, STF-083010 markedly mitigated CCl_4_-induced upregulation of hepatic XBP1s/XBP1t (Figure 1B). Additionally, we found that STF-083010 had no effect on ER stress marker protein GRP78, which further demonstrated that STF-083010 was a targeted inhibitor of IRE1α RNase activity (Figure 1C). The results demonstrated that STF-083010 can selectively inhibit CCl_4_-induced hepatic IRE1α RNase activation in the study.

### STF-083010 Mitigates Liver Injury and Inflammation in CCl_4_-Treated Mice

**FIGURE 2:**
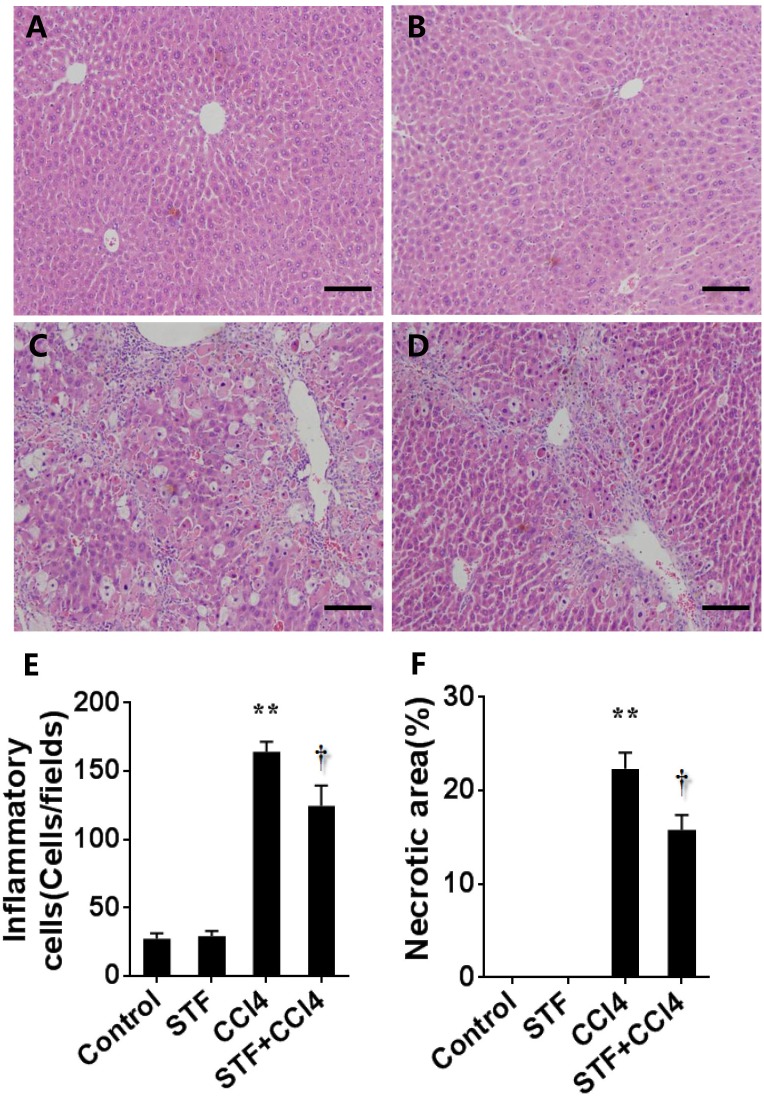
Effects of STF-083010 (STF) on liver injury and inflammation following long-term CCl_4_ treatment. Mice were i.p. injected with CCl_4_ (0.15 ml/kg, twice per week). In STF-083010+CCl_4_ group, mice were injected with STF-083010 (30 mg/kg, i.p.), twice per week, beginning from the 6th week after CCl_4_ injection. All mice were sacrificed after 8 weeks with CCl_4_ treatment. Representative hepatic histological photomicrographs in mice of control group **(A)**, STF group **(B)**, CCl_4_ group **(C)**, and combination of STF plus CCl_4_ group **(D)** are shown in the picture (H&E, magnification ×100). Scale bars are 100 μm. The number of inflammatory cells **(E)** was counted in 12 randomly selected fields from each slide. **(F)** Morphometric analysis was performed to evaluate the percentage of necrotic site in each section. All data were expressed as means ± SEM (*n* = 6). ^∗∗^*P* < 0.01 versus control group. ^†^*P* < 0.05 versus CCl_4_ group.

**FIGURE 3:**
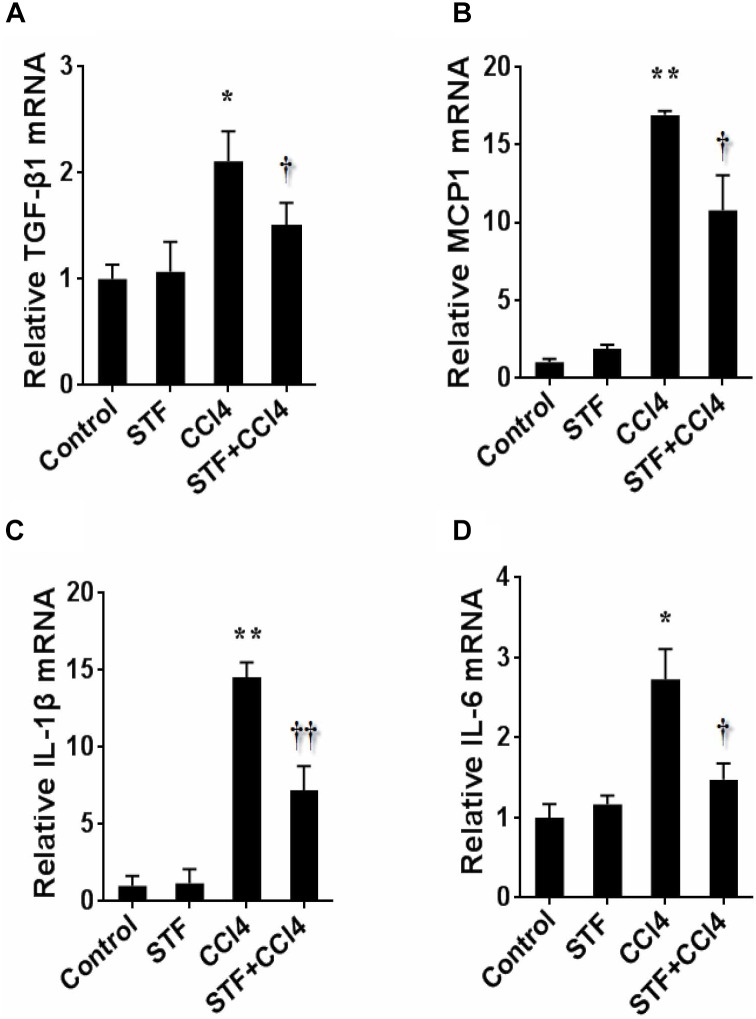
Effects of STF-083010 (STF) on hepatic inflammatory cytokines following long-term CCl_4_ administration. Mice were i.p. injected with CCl_4_ (0.15 ml/kg, twice per week). In STF-083010+CCl_4_ group, mice were injected with STF-083010 (30 mg/kg, i.p.), twice per week, beginning from the 6th week after CCl_4_ injection. All mice were sacrificed after 8 weeks with CCl_4_ treatment. Hepatic TGF-β1 **(A)**, MCP1 **(B)**, IL-1β **(C)**, and IL-6 **(D)** mRNAs were measured using real-time RT-PCR. All data were expressed as means ± SEM (*n* = 6). ^∗^*P* < 0.05, ^∗∗^*P* < 0.01 versus control group. ^†^*P* < 0.05, ^††^*P* < 0.01 versus CCl_4_ group.

As shown in Table 2, long-term CCl_4_ treatment caused a significant increase in liver weight, however, STF-083010 had no effect on liver weight in mice. This study then analyzed the effect of STF-083010 on serum biological parameters. Certainly, CCl_4_ treatment markedly enhanced the levels of serum ALT, TBIL, DBIL, and TBA, and STF-083010 had obviously extenuated CCl_4_-induced exaltation of ALT, DBIL, and TBA levels (Table 2). The effects of STF-083010 on hepatic histopathological damage were presented in Figure 2. Indeed, a large amount of inflammatory cells infiltration around necrotic tissue were observed in CCl_4_-treated liver tissue sections. Interestingly, STF-083010 manifestly mitigated CCl_4_-induced hepatic necrosis and inflammation (Figures 2C–F). Moreover, this study also analyzed the effect of STF-083010 on CCl_4_-induced inflammatory cytokines expression. Certainly, CCl_4_ administration obviously improved the levels of hepatic transforming growth factor beta (TGF-β1), monocyte chemotactic protein 1 (MCP1), interleukin-1 (IL-1β) and IL-6 mRNAs. Of interest, STF-083010 apparently attenuated CCl_4_-induced upregulation of hepatic TGF-β1, MCP1, IL-1β, and IL-6 mRNAs (Figure 3). The results illustrated that STF-083010 obviously extenuated liver injury and inflammation.

**Table 2:** Physiologic and serum parameters.

Parameters	Control (*n* = 11)	STF-083010 (*n* = 11)	CCl_4_ (*n* = 11)	STF-083010+CCl_4_ (*n* = 10)
Liver weight (g)	1.72 ± 0.26	1.82 ± 0.08	2.56 ± 0.11^∗∗^	2.59 ± 0.03
Liver to body Weight ratio (%)	4.54 ± 0.11	4.71 ± 0.10	6.41 ± 0.20^∗∗^	6.47 ± 0.10
ALT (U/L)	37.83 ± 4.92	45.00 ± 2.58	313.45 ± 22.76^∗∗^	237.14 ± 22.51^†^
TBIL (μmol/l)	3.83 ± 0.14	4.12 ± 0.55	7.87 ± 0.61^∗∗^	7.84 ± 0.63
DBIL (μmol/l)	0.67 ± 0.04	0.70 ± 0.18	1.42 ± 0.15^∗∗^	0.77 ± 0.20^†^
TBA (μmol/l)	4.01 ± 1.88	3.16 ± 1.22	13.51 ± 1.65^∗∗^	5.02 ± 1.20^††^

*^∗∗^P < 0.01 versus control group; ^†^P < 0.05, ^††^P < 0.01 versus CCl_4_ group. Measurement data were expressed as means ± SEM. ALT, alanine aminotransferase; TBIL, total bilirubin; DBIL, direct bilirubin; TBA, total bile acids.*

### STF-083010 Reduces the Expression of Hepatic α-SMA in CCl_4_-Treated Mice

**FIGURE 4:**
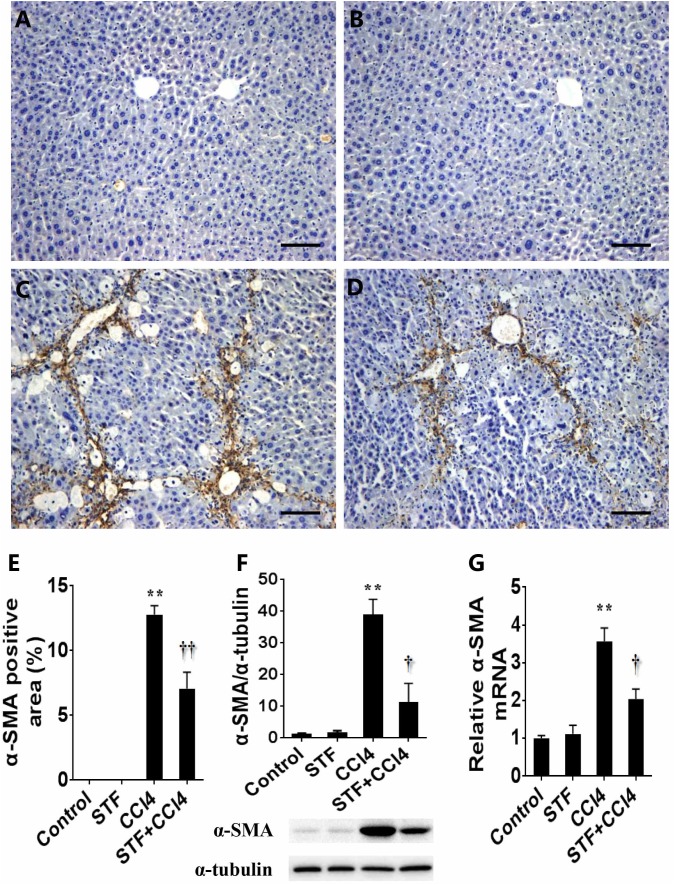
Effects of STF-083010 (STF) on CCl_4_-induced expression of hepatic α-SMA. Mice were i.p. injected with CCl_4_ (0.15 ml/kg, twice per week). In STF-083010+CCl_4_ group, mice were injected with STF-083010 (30 mg/kg, i.p.), twice per week, beginning from the 6th week after CCl_4_ injection. All mice were sacrificed after 8 weeks with CCl_4_ treatment. Representative liver tissue sections stained with immunohistochemistry for α-SMA (**A**, control; **B,** STF; **C**, CCl_4_; and **D**, STF+CCl_4_, magnification × 100). Scale bars are 100 μm. **(E)** Morphometrical analysis was implemented for assessing percentages of a-SMA positive area in each section. **(F)** Hepatic α-SMA protein was measured using immunoblot. Quantitative analyses of scanning densitometry for α-SMA on six samples from six different mice were carried out. **(G)** Hepatic α-SMA mRNA was measured using real-time RT-PCR. All data were expressed as means ± SEM (*n* = 6). ^∗∗^*P* < 0.01 versus control group. ^†^*P* < 0.05, ^††^*P* < 0.01 versus CCl_4_ group.

The effect of STF-083010 on hepatic α-SMA, a marker related to HSCs activation, was analyzed. Immunohistochemistry results showed that the majority of hepatic α-SMA was distributed in the bridging fibrosis area (Figure 4C), but hepatic α-SMA was almost no distributed in control group (Figure 4A) and STF-083010 alone group (Figure 4B). Further analysis illustrated that the percentages of hepatic α-SMA positive area in CCl_4_-treated mice were obviously enhanced (Figure 4E). Western blot showed that CCl_4_-induced hepatic α-SMA protein was evidently upregulated in mice (Figure 4F). Interestingly, STF-083010 evidently attenuated the elevation of hepatic α-SMA protein during the CCl_4_-induced liver fibrosis (Figures 4D–F). Correspondingly, long-term CCl_4_ treatment obviously raised the expression of hepatic α-SMA mRNA. Of interest, STF-083010 markedly inhibited CCl_4_-induced up-regulation of hepatic α-SMA mRNA (Figure 4G).

### STF-083010 Extenuates CCl_4_-Induced Liver Injury and Subsequent Liver Fibrosis

**FIGURE 5:**
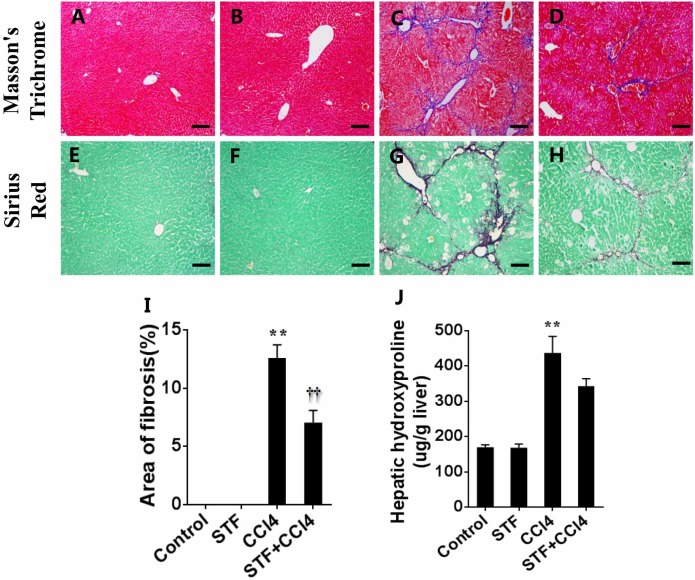
Effects of STF-083010 (STF) on CCl_4_-induced liver injury and liver fibrosis. Mice were i.p. injected with CCl_4_ (0.15 ml/kg, twice per week). In STF-083010+CCl_4_ group, mice were injected with STF-083010 (30 mg/kg, i.p.), twice per week, beginning from the 6th week after CCl_4_ injection. All mice were sacrificed after 8 weeks with CCl_4_ treatment. **(A–H)** Representative liver tissue sections stained with masson’s trichrome and sirius red for collagen. A/E (control), B/F (STF), C/G (CCl_4_), and D/H (combination of STF plus CCl_4_) are shown (magnification × 100). Scale bars are 100 μm. **(I)** Morphometric analysis was performed to evaluate the percentage of specific collagen fibers in each section. **(J)** Hepatic hydroxyproline was measured. All data were expressed as means ± SEM (*n* = 6). ^∗∗^*P* < 0.01 versus control group. ^††^*P* < 0.01 versus CCl_4_ group.

Liver fibrosis was determined using masson’s trichrome staining and sirius red staining. Indeed, obvious bridging fibers were observed in the liver of CCl_4_-treated mice (Figures 5C,G), but almost no observed in control group (Figures 5A,E) and STF-083010 alone group (Figures 5B,F). STF-083010 evidently extenuated liver injury and subsequent liver fibrosis in mice (Figures 5D,H). Morphological analysis demonstrated that fibrotic area was markedly decreased in mice treated with STF-083010 plus CCl_4_ versus CCl_4_ alone (Figure 5I). The effect of STF-083010 on hepatic hydroxyproline (Hyp), an indicator of liver fibrosis, was analyzed. CCl_4_-induced hepatic Hyp content was markedly enhanced in mice, and STF-083010 reduced CCl_4_-induced exaltation of hepatic Hyp content (Figure 5J).

### Effects of STF-083010 on CCl_4_-Induced Expression of Hepatic MiR-122 and Its Target Genes

**FIGURE 6:**
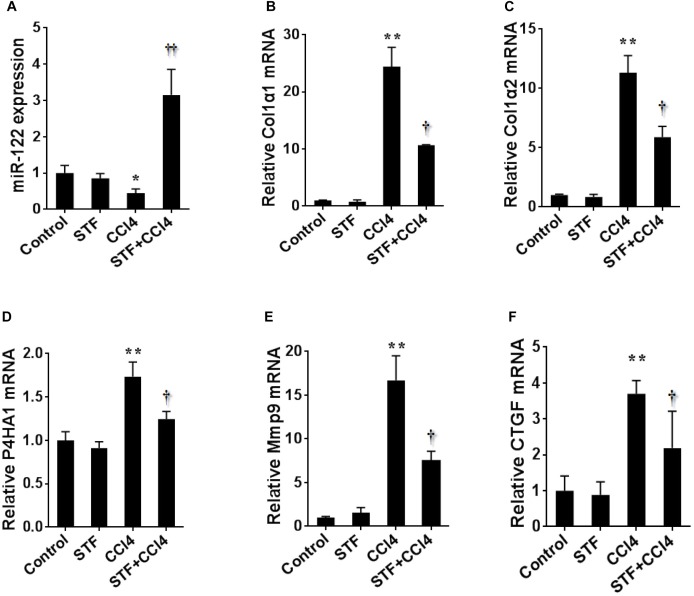
Effects of STF-083010 (STF) treatment on CCl_4_-induced expression of hepatic miR-122 and its target genes. Mice were i.p. injected with CCl_4_ (0.15 ml/kg, twice per week). In STF-083010+CCl_4_ group, mice were injected with STF-083010 (30 mg/kg, i.p.), twice per week, beginning from the 6th week after CCl_4_ injection. All mice were sacrificed after 8 weeks with CCl_4_ treatment. Hepatic miR-122 and its target genes related with collagen maturation and ECM production, such as Col1α1, Col1α2, P4HA1, Mmp9, and CTGF mRNAs were measured using real-time RT-PCR. **(A)** miR-122; **(B)** Col1α1; **(C)** Col1α2; **(D)** P4HA1; **(E)** Mmp9; **(F)** CTGF. All data were expressed as means ± SEM (*n* = 6). ^∗^*P* < 0.05, ^∗∗^*P* < 0.01 versus control group. ^†^*P* < 0.05, ^††^*P* < 0.01 versus CCl_4_ group.

To further study the effect of STF-083010 on miR-122 in liver fibrosis, this study examined the effect of STF-083010 on the expression level of hepatic miR-122 using real-time RT-PCR. The repeated CCl_4_ administration led to evident downregulation of hepatic miR-122 in mice Figure 6A). Conversely, the CCl_4_-induced hepatic miR-122 could be significantly raised by STF-083010. Indeed, the downstream target genes of miR-122, such as synthesis of collagen (Col1α1, Col1α2, and P4HA1), matrix metallopeptidase (Mmp9), connective tissue growth factor (CTGF) (Figures 6B–F) and inflammatory factors (TGF-β1, MCP1, and IL-1β) (Figure 3) mRNAs levels, were upregulated in CCl_4_-induced fibrotic mice. Interestingly, this study also found that STF-083010 obviously elevated the level of miR-122 expression and decreased its target genes related with collagen maturation and ECM production.

## Discussion

Under conditions of ER stress, IRE1α RNase is activated through dimerization and autophosphorylation and removes 26 nucleotides from unspliced X-box binding protein 1 (XBP1u) mRNA to generate XBP1s, producing a functional XBP1s transcription factor (Chen and Brandizzi, 2013). A few studies recently reported that IRE1α RNase activation was involved in fibrosis by splicing XBP1 mRNA (Mo et al., 2015; Heindryckx et al., 2016). However, it remains unclear whether the mechanism of independent IRE1α RNase cleaving XBP1 mRNA is involved in liver injury and liver fibrosis. Additionally, a recent study showed that STF-083010 could efficiently correct nonalcoholic fatty liver disease (NAFLD) by limiting IRE1α signaling (Lebeaupin et al., 2018). Repeated CCl_4_ stimulation can lead to persistent liver injury and subsequent liver fibrosis. In this study, the effect and possible mechanism of IRE1α RNase inhibitor STF-083010 on CCl_4_-induced liver injury and subsequent liver fibrosis were explored in mice. As expected, pharmacological inhibition of the chemical small molecule STF-083010 on IRE1α RNase activity markedly extenuated CCl_4_-induced liver injury and thus protected against liver fibrosis in mice.

It was generally believed that the main cell event in the progression of liver fibrosis was the activation of HSCs (Chen et al., 2015). In fact, the studies showed that α-SMA was a marker of the initiation stage of HSCs activation, which was evidently raised in the liver region of bridging fibrosis, and the mRNA levels of hepatic col1α1 and col1α2, markers for the perpetuation stage of HSCs activation, were clearly elevated in fibrotic mice (Osterreicher et al., 2009; Wang et al., 2013; Heindryckx et al., 2016). Several reports demonstrated that inflammatory cytokines such as MCP1, IL-1β, IL-6, and TGF-β1 were closely related to the activation of HSCs (Moles et al., 2014; Wu et al., 2015; Kim et al., 2017; Kiagiadaki et al., 2018). It was reported that pre-treatment with IRE1α inhibitor reduced pro-inflammatory cytokines production in tumor necrosis factor (TNF)-receptor-associated periodic fever syndrome (TRAPS) dermal fibroblasts (DFs) (Harrison et al., 2018). On the other hand, these inflammatory cytokines were also the downstream target genes of miR-122 (Hsu et al., 2012; Nakamura et al., 2015; Yin et al., 2016; Tang et al., 2017). In the present study, the result showed that STF-083010 evidently inhibited the upregulation of hepatic α-SMA, Col1α1 and Col1α2 in mice, but these also may be attributed to HSCs expression and the fact that STF-083010 alleviates liver injury by inhibiting CCl_4_-mediated hepatocyte death. Additionally, the study found that CCl_4_-upregulated the mRNA levels of hepatic MCP1, IL-1β, IL-6, and TGF-β1 were attenuated by STF-083010. These results probably suggest that STF-083010 regulates collagen production or degradation (Mmp9) by inhibiting subsequent inflammatory response.

MiRNAs are short (21–24 nucleotides), noncoding RNA molecules that interfere with gene expression at the posttranscriptional level by inducing mRNA degradation or blocking gene translation, which, in turn, decreases or prevents protein synthesis (Bartel, 2009; Liu et al., 2014; Murakami and Kawada, 2017). It was reported that IRE1α was capable of degrading miRNAs, in addition to its well-known capability to degrade mRNA, which provided a new probable connection between IRE1α and miRNAs (Upton et al., 2012; Heindryckx et al., 2016). MiRNAs had been shown to play an important regulatory role in the pathogenesis and treatment of progressive liver injury including NAFLD and liver fibrosis (Leti et al., 2015; Liu et al., 2016; Su et al., 2018). Importantly, miR-122 was the high specificity expression accounted for more than 70% of total miRNAs in the adult liver, and a central player in liver biology and disease, which become attractive therapeutic targets (Girard et al., 2008; Hayes and Chayama, 2016; Matsuura et al., 2016). The function of miR-122 was hepatic development, differentiation, homeostasis and metabolism, and was closely related to important liver diseases, such as lipid metabolism, acute liver injury, cirrhosis and HCC (Esau et al., 2006; Xu et al., 2010; Kim et al., 2011; Bandiera et al., 2015; Leelahavanichkul et al., 2015). Numerous studies demonstrated that hepatic miR-122 expression was significantly decreased in liver disease and correlated with the degree of liver damage (Tsai et al., 2012; Trebicka et al., 2013; Csak et al., 2015). Recently, a research group investigated that miR-122 negatively correlated with liver fibrosis as detected by histology and FibroScan (Halasz et al., 2015). Additionally, miR-122 regulated collagen production via targeting HSCs and suppressing prolyl-4-hydroxylase alpha polypeptide I (P4HA1) expression (Li et al., 2013). Indeed, the present study determined that repeated CCl_4_ administration led to obvious downregulation of hepatic miR-122 in mice, and the effect was markedly reversed by STF-083010. This result was probably due to the significant inhibition of IRE1α RNase activity by STF-083010, which prevented it from cleavage by miR-122. However, its specific mechanism will be further explored. In addition, the study demonstrated that the downstream target genes of miR-122, such as Col1α1, Col1α2, CTGF, P4HA1, and Mmp9 mRNAs, were negatively correlated with the expression level of miR-122 in mouse liver. These may be due to STF-083010 alleviates CCl_4_-induced liver damage and inflammation. This study may reveal a new mechanism by which IRE1α RNase controls liver injury and subsequent liver fibrosis associated with hepatic miR-122 and its target genes, but it still needs to be confirmed further.

ER stress and oxidative stress are interacted states that can occur in cells as part of normal physiology (Batool et al., 2018; Chen et al., 2018). The results of this study found that the expression level of 3-NT (a marker of oxidative stress) was evidently increased in CCl_4_ model group, and IRE1α RNase inhibitor STF-083010 can slightly reduce oxidative stress (please refer to Supplementary Figure S1). However, its specific mechanism remains to be further explored. Notably, study has reported that IRE1α RNase specific inhibitor 4μ8C is also a potent cellular antioxidant (Chan et al., 2018). Accordingly, the chemical small molecule STF-083010 may also have potential antioxidant effects, which will be further explored in subsequent studies.

On the other hand, to investigate the effect of STF-083010 on mitochondria-associated proteins, we briefly examined the expression levels of mitochondrial heat shock proteins HSPA9 (also known as GRP75, PBP74), HSPD1 and mitochondrial membrane proteins (mitofusin-1/-2, MFN1, and MFN2). These proteins play essential roles in the control of cell proliferation, facilitate the correct folding and assembly of imported proteins and mediate mitochondrial outer membrane fusion (Kaul et al., 2002; Cappello et al., 2014; Zorzano et al., 2015). In the present study, the result showed that hepatic HSPA9 and HSPD1 mRNAs were significantly upregulated in CCl_4_-treated mice, but STF-083010 had no effect on their expression. Additionally, long-term CCl_4_ treatment did not significantly affect the expression levels of hepatic MFN1 and MFN2 mRNAs (please refer to Supplementary Figure S2). The specific mechanism will be further explored.

The present study found that STF-083010 inhibited CCl_4_-induced liver injury and thus also liver fibrosis in mice. Namely, subsequent observed results were due to the hepatoprotective effect of STF-083010. The results also demonstrated that IRE1α RNase was a potential target of therapy and STF-083010 might be an effective pharmacological agent of chemical therapy in liver fibrosis. However, this study has several limitations. Firstly, the study did not prove the effects of STF-083010 on other models of liver fibrosis. Secondly, the present study did not determine the exact mechanism of IRE1α RNase cleave miR-122. Thirdly, it should be further explored how miR-122 produced in hepatocyte mediated its target gene involved in liver fibrogenesis in HSCs. Additionally, further studies need to define a dose response curve to evaluate the effects of different doses STF-083010 on liver fibrosis.

In summary, the present study illustrated that STF-083010, an IRE1α RNase specific inhibitor, alleviated CCl_4_-induced liver injury and thus protected against liver fibrosis. Furthermore, the present study found that STF-083010 also had an effect on oxidative stress. Mechanistic exploration found that STF-083010 markedly reversed downregulation of hepatic miR-122 in CCl_4_-induced liver fibrosis. Correspondingly, STF-083010 inhibited CCl_4_-induced upregulation of miR-122 target genes. This study provided partial evidence that hepatic IRE1α RNase was involved in CCl_4_-induced liver fibrosis associated with hepatic miR-122. Thus, this study illustrated that IRE1α RNase specific inhibitor STF-083010 might be a potential therapy strategy for patients with progressive fibrotic diseases.

## Author Contributions

J-QW and CZ designed the research. Q-QC, CZ, M-QQ, and JL performed the research. J-QW, CZ, HW, and D-XX contributed new reagents or analytic tools. Q-QC and CZ analyzed the data. Q-QC wrote the paper. J-QW, CZ, and D-XX revised the paper.

## Conflict of Interest Statement

The authors declare that the research was conducted in the absence of any commercial or financial relationships that could be construed as a potential conflict of interest.

## Footnotes

1 http://rsb.info.nih.gov/ij/

## Abbreviations

3-NT: 3-Nitrotyrosine
α-SMA: α-smooth muscle actin
Col1α: collagen I alpha
CTGF: connective tissue growth factor
ER: endoplasmic reticulum
HSPA9: heat shock protein family A (Hsp70) member 9
HSPD1: heat shock protein family D (Hsp60) member 1
IL-1β: interleukin-1 beta
IRE1α: inositol-requiring enzyme 1 alpha
MCP1: monocyte chemoattractant protein-1
MFN-1/-2: mitofusin-1/-2
Mmp9: matrix metalloproteinase-9
P4HA1: prolyl-4-hydroxylase alpha polypeptide I
TGF-β1: transforming growth factor beta-1
XBP1s: spliced X-box binding protein 1
XBP1t: total X-box binding protein 1.
