# Supplementary material for: Inositol-Requiring Enzyme 1 Alpha Endoribonuclease Specific Inhibitor STF-083010 Alleviates Carbon Tetrachloride Induced Liver Injury and Liver Fibrosis in Mice
Source: Front Pharmacol. 2018 Nov 27;9:1344. doi: 10.3389/fphar.2018.01344 (PMC6277551; doi:10.3389/fphar.2018.01344)
Supplement: Supplementary file 1 [file Data_Sheet_1.PDF]

Supplemental Information

Inositol-Requiring Enzyme 1 Alpha Endoribonuclease Specific Inhibitor STF-083010 Alleviates Carbon Tetrachloride Induced Liver Injury and Liver Fibrosis in Mice

Qian-Qian Chen [1,#], Cheng Zhang [3,#], Ming-Qiang Qin [1], Jian Li [3], Hua Wang [3], De-Xiang Xu [3 *], Jian-Qing Wang [1, 2 *]

[1] The Second Affiliated Hospital, Anhui Medical University, Hefei 230601, China

[2] The Fourth Affiliated Hospital, Anhui Medical University, Hefei 230022, China

[3] Department of Toxicology, Anhui Medical University, Hefei 230032, China

***Corresponding author:**

Jian-Qing Wang, jianqingwang81@126.com

De-Xiang Xu, xudex@126.com

[#] These authors contributed equally to this work.

Figure S1

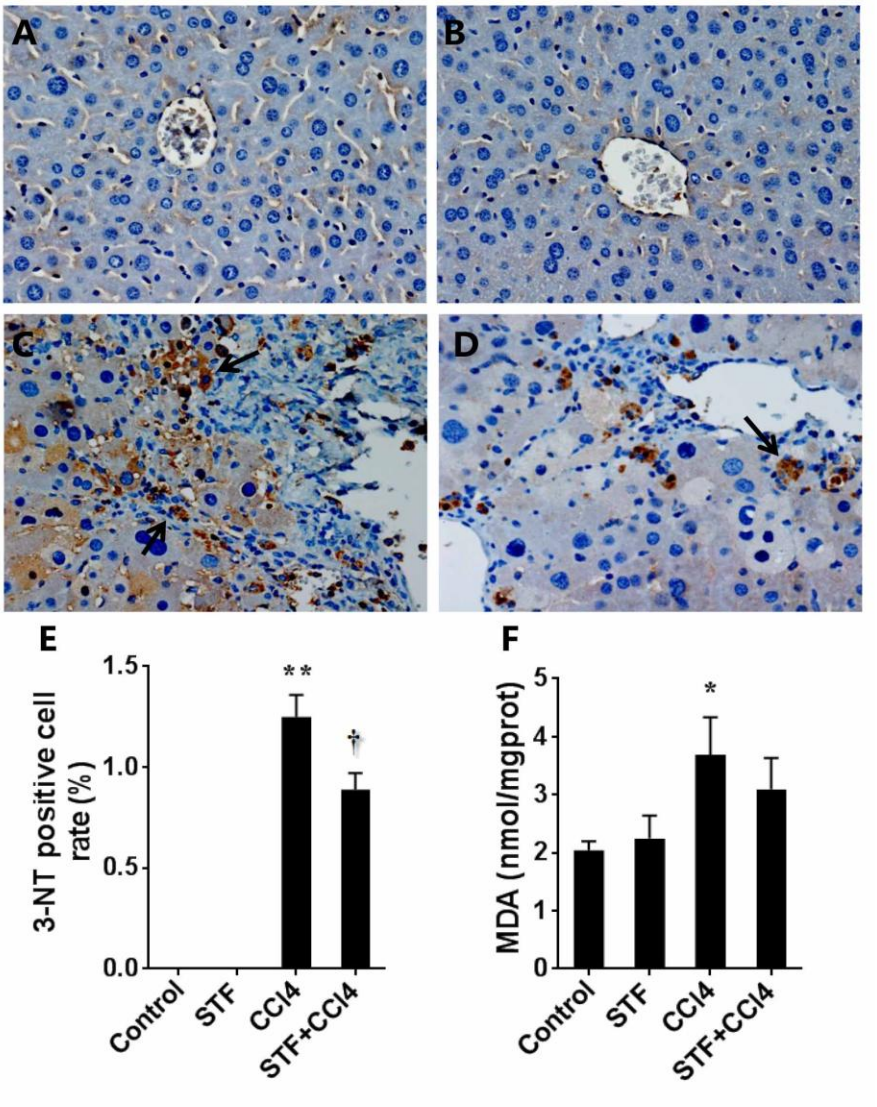

FIGURE S1. Effect of STF-083010 (STF) on CCl_4-induced oxidative stress in mice

Mice were i.p. injected with CCl_4 (0.15 ml/kg, twice per week). In STF-083010+CCl_4 group, mice were injected with STF-083010 (30 mg/kg, i.p.), twice per week, beginning from the sixth week after CCl_4 injection. All mice were sacrificed after 8 weeks with CCl_4 treatment. Representative liver tissue sections were stained with immunohistochemistry for 3-NT (a marker of oxidative stress) (A. control, B. STF, C. CCl_4 and D. STF+CCl_4, magnification × 400). (E) Morphometrical analysis was implemented for assessing the number of 3-NT positive staining cells in each section. (F) Hepatic malondialdehyde (MDA) was measured. All data were expressed as means ± SEM (n = 6). **P* < 0.05, ***P* < 0.01 versus control group. †*P* < 0.05, ††*P* < 0.01 versus CCl_4 group.

Figure S2

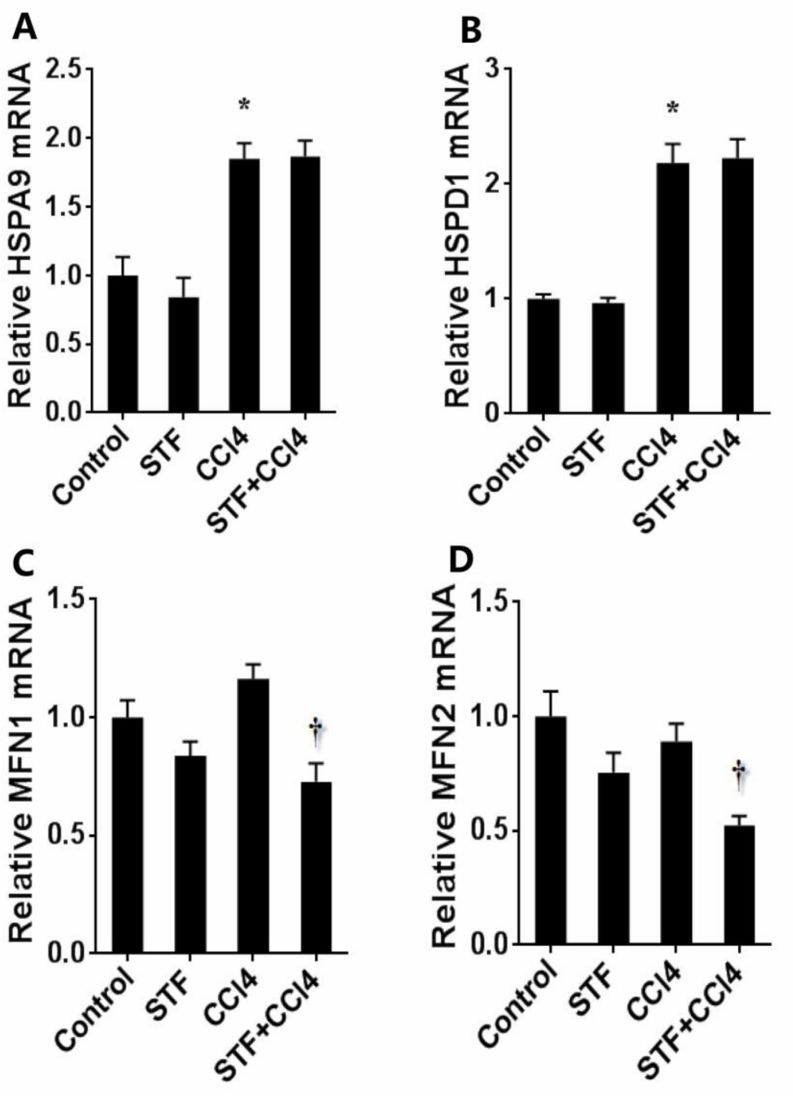

FIGURE S2. Effect of STF-083010 (STF) on CCl_4-induced the expression of hepatic mitochondria-associated genes in mice

Mice were i.p. injected with CCl_4 (0.15 ml/kg, twice per week). In STF-083010+CCl_4 group, mice were injected with STF-083010 (30 mg/kg, i.p.), twice per week, beginning from the sixth week after CCl_4 injection. All mice were sacrificed after 8 weeks with CCl_4 treatment. The expression levels of hepatic mitochondrial heat shock proteins HSPA9 (also known as GRP75, PBP74), HSPD1 and mitochondrial membrane proteins (mitofusin-1/-2, MFN1 and MFN2) mRNAs were measured using real-time RT-PCR. (A) HSPA9; (B) HSPD1; (C) MFN1; (D) MFN2. All data were expressed as means ± SEM (n = 6). *$P < 0.05$, **$P < 0.01$ versus control group. †$P < 0.05$, ††$P < 0.01$ versus CCl_4 group.
